# Exploration on the scientific rationality of low-carbon investment projects from the perspective of supply chain: a case study of CS thermal power plant

**DOI:** 10.1007/s11356-023-29602-6

**Published:** 2023-09-14

**Authors:** Zhen Li, Yushi Tian, Shenglan Li, Zhuoyu Huo, Hua Zhang

**Affiliations:** 1 School of Accounting, Hunan University of Finance and Economics, Changsha, China; 2 School of Business, Central South University, Changsha, China; 3 Institute of Innovation and Entrepreneurship, Loughborough University, London, UK

**Keywords:** Low-carbon supply chain, Thermal power plant, Project investment evaluation

## Abstract

Climate warming has gradually become a major problem threatening human survival, and countries have begun to pay attention to carbon emissions. Energy conservation and emission reduction has become a central task in China’s economic development since the 14th Five-Year Plan. As the main force of carbon emissions in China, thermal power industry is bound to become the focus of attention in China’s low-carbon development strategy and energy conservation and emission reduction. Moreover, with the marketization of the power industry, the state has joined the market competition at the power generation sectors and the power sale sectors, and implemented the “opening the middle of the two pipes.” Therefore, the coverage of influence of carbon emissions and carbon investment behavior of power generation companies is not limited to itself, but will also be extended to the supply chain level. Based on the above background, this paper evaluates the scientific rationality of low-carbon investment projects of thermal power enterprises from the perspective of low-carbon supply chain, which not only can help enterprises achieve a win–win situation of economic and environmental benefits, but also contribute to the carbon emission reduction of the entire supply chain, thereby promoting China’s entire social and economic energy conservation and emission reduction work.

## Introduction

With the acceleration of economic globalization, global warming caused by greenhouse gases, mainly carbon dioxide, is becoming increasingly serious. Since the British government issued the “Energy White Paper” in the early twenty-first century, which proposed the “low-carbon economy,” mankind has gradually developed a deeper understanding of environmental issues and economic development. The Copenhagen Accord adopted by the United Nations at the Climate Conference marked the global entry into a low-carbon economy era. The core concepts of “high efficiency, high efficiency, high efficiency” and “low energy consumption, low pollution, and low emissions” have put forward new requirements for the development of enterprises. In particular, production enterprises should not blindly pursue economic benefits during the operation process, but should be based on the diversity goals and sustainable development concept of social construction. During project investment evaluation, attention should be paid to reducing carbon emissions, and efforts should be made to achieve the unity of economic, social, and environmental benefits.

As the largest developing country in the world, China’s carbon dioxide emissions have ranked first in the world, seriously restricting the development of the domestic economy, bringing pressure to the world environment, and posing a threat to the survival and development of mankind. Since the “14th Five Year Plan,” the government has made energy conservation and emission reduction a central task of economic development. Although many achievements have been made, the carbon dioxide emissions of the power industry still account for a high proportion, almost half of the national carbon dioxide emissions, and for a long time, the structure of power sources, mainly thermal power generation, will not change. Therefore, the power industry has become a key industry in promoting low-carbon development strategies and focusing on energy conservation and emission reduction in China. Currently, the goal of China’s electricity industry is to achieve marketization, and the investment evaluation of low-carbon projects still adopts the traditional method, focusing on financial indicators, without considering the impact of carbon factors. However, in the research of carbon management accounting, it is only at the conceptual stage of proposing to establish an investment evaluation system, and cannot accurately make an evaluation. If the relevant theoretical basis of the low-carbon supply chain is matched with its investment project evaluation, combined with the current development status of power generation enterprises, and the characteristics of the power industry are comprehensively considered, a set of low-carbon project investment evaluation system from a supply chain perspective is established to accurately evaluate the achievements of low-carbon project investment in the low-carbon supply chain of power generation enterprises. It can help enterprises and even the entire industry improve their sustainable development ability in the investment field.

Taking CS thermal power plants as an example, this paper conducts a multi-level analysis of the characteristics of low-carbon project investment evaluation based on the low-carbon supply chain theory. Based on the financial judgment indicators in traditional investment evaluation analysis, carbon factor indicators are added, and indicators related to the development of the supply chain are selected through expert scoring, theoretical analysis, and other methods. Subsequently, a multi-level low-carbon project investment evaluation index system is constructed based on the DPSIR model framework. Select appropriate model methods to form a low-carbon project investment evaluation system for the supply chain with thermal power plants as the core, and apply the system to the low-carbon project investment plans of CS thermal power plants. Evaluate and score the three alternative low-carbon project investment plans, and select the plan with the highest total benefit to help CS thermal power plants make the optimal choice before investment.

## Review

### Low-carbon economy

The concept of low carbon was first put forward by KINZIG.A.P in the 1990s. The so-called low carbon refers to reducing the emission of greenhouse gases mainly containing carbon dioxide to a low level. Most western scholars focus on the relationship between carbon emissions and economic growth and per capita income.

Bezdek 1993 pointed out that in the past, people believed that environmental regulation was often not conducive to the development of the traditional economy and could not provide advantages in international competition (Nawaz et al. 2021). Although this view can help enterprises achieve rapid development in the short term, it is not conducive to the sustainable development of the overall economy in the future. Grossman and Krueger 1991 studied the relationship between economic growth and air quality in 42 countries and regions, and found an inverted U-shaped relationship between economic growth and air pollution (Su et al. 2021). Holtz-Eakin and Selden 1995 used global data to study the relationship between economic growth and carbon dioxide emissions, and concluded that the marginal emission tendency of carbon dioxide decreases with economic growth (Lv and Li 2021). Zhongyu and Zhongxiang (2021) carefully analyzed the shortcomings of the whole process of China’s low-carbon economic development, deeply explored its development trend management system, and examined the continuous impact of low-carbon economy on world rights and interests from the perspective of the continuous impact of low-carbon economy on world rights and interests (Wang et al. 2020). Wang et al. (2022) pointed out that many countries have successively proposed carbon neutrality goals, leading to increased competition in the international low-carbon economy, and low-carbon economic development has become a global irreversible trend (Nasir et al. 2022).

### Project investment evaluation

Project investment evaluation refers to the use of system principle, investment optimization principle, risk uncertainty principle, and so on to make a comprehensive and comprehensive evaluation of investment projects, so that a certain amount of capital investment to obtain the maximum output activities. In the case of low-carbon concept gradually popular, enterprises began to consider low-carbon environmental benefits, not just economic benefits, when evaluating project investment benefits.

When investing in low-carbon projects, domestic and foreign scholars use different indicators to evaluate the project. Cararo et al. (2012) studied the changes in corporate energy investment caused by the green low-carbon economy by applying a comprehensive evaluation model (Doğan et al. 2021). Matsumoto et al. (2018) constructed a single-stage optimization decision model for corporate emission reduction under the carbon emission trading mechanism, and on this basis, four emission reduction approaches were given (Wang et al. 2019a). Dietz and Hepburn (2013) confirmed that the traditional cost–benefit analysis method is no longer applicable in the context of climate change and energy policy changes by applying global carbon emission reduction targets and new energy project construction cases. Based on the compound policy situation of carbon trading and carbon tax (Xiao et al. 2022), Tong et al. (2022) constructed a two-level planning decision model of carbon emission reduction including government and enterprises (Baskaya et al. 2022).

### Low-carbon supply chain

Low-carbon supply chain is to integrate low-carbon and environmental protection thinking into the whole supply chain, minimize the cost of theme activities, form a complete low-carbon supply chain system from the procurement of raw materials and other resources to industrial manufacturing and delivery, and achieve a win–win situation between economy and environment.

On how to reduce carbon emissions in the supply chain, Larsen et al. (2012) found that carbon emissions in most industries are located upstream of the supply chain and can be reduced by tax (Li et al. 2021). Wang et al. (2019b) found that in the supply chain, governments setting appropriate carbon quotas can motivate enterprises to invest in low-carbon technologies, thereby reducing carbon emissions (Montefrio and Dressler 2016). Regarding the energy conservation and emission reduction strategy of the power supply chain, Ford (2008) believed that the construction of clean energy power generation can effectively reduce the carbon emissions of the power supply chain (Herman and Shenk 2021), and further explored the role and impact of carbon sequestration technology in the carbon emission reduction of the power industry chain. Flynn et al. (2008) explored the carbon footprint measurement in the power supply chain with the power industry as the research background (Kouhizadeh et al. 2019). Adeoye and Spataru (2019) found that the demand side of the power industry has great energy-saving potential, and its management can make a greater contribution to carbon emission reduction (Yu et al. 2021).

## Analysis of the investment status of CS thermal power plant project

### Current status of energy consumption and waste emissions in the power supply chain

#### Current status of energy consumption and waste discharge in the raw material extraction process

Due to the characteristics of CS thermal power plant, the main raw material in the production process is coal. Upstream A coal mining enterprise will generate some carbon emissions during the mining process, mainly including three types: direct and indirect carbon emissions caused by the consumption of raw coal, gasoline, and electricity during the mining process; direct emissions caused by the escape of gases such as CH4 and CO2 adsorbed in the coal; and direct emissions caused by uncontrolled oxidation and spontaneous combustion of raw coal and coal gangue.

In 2018, A coal mining enterprise produced 3029.61 million tons of raw coal. According to data statistics, the total carbon emissions in 2018 were 570,784.67 tons, with a unit output value of 10.6 tons/10,000 yuan and a unit output value of 0.168 tons/ton. The annual coal consumption of CS thermal power plant is approximately 2.6 million tons.

Upstream A coal mining enterprise will cause a load on the environment during the mining process, resulting in external damage costs to the forest. This part of the cost is borne by A coal mining enterprise, and the accounting basis for external damage costs in this link is shown in Table 1. Table 2 shows the main economic indicators of thermal power plant production in 2018. Table 3 shows that the energy input table of CS thermal power plant. Table 4 defines energy output of CS thermal power plant.

**Table 1:** Coal mining cost accounting basis

Accounting items	Value
Disturbed topsoil	1.10 tons/ton of coal
Average bulk density of soil	1.70 tons/cubic meter
Soil layer thickness	0.30 m
Forest value	24,900 yuan/hectare

**Table 2:** Main economic indicators of thermal power plant production in 2018

Year	Generation	Consumption rate	Fuel consumption	Coal consumption
2018	564.00 million MWH	5.69%	156.94 tons	293.70 g/kwh

**Table 3:** Energy input table of CS thermal power plant

Name	Unit	Number
Coal	10,000 tons	260.00
Diesel Oil	Tons	156.94
Electricity	10,000 KW/H	6000.00

**Table 4:** Energy output of CS thermal power plant

Name	Unit	Number
Electricity	10,000 MWH	564.00
Energy Loss	10,000 Yuan	4074.1

The annual coal consumption of CS thermal power plant is about 2.6 million tons, so the external damage cost is calculated as follows (unit, million yuan):

$${({1.1}^\ast260^\ast10^4)}^\ast{2.49}^\ast10{}^{-4}/({1.7}^\ast0.3)=1396.35$$

During the coal transportation process, A coal mining enterprise is located in Shanxi, and the coal is mainly transported by railway to the power plant. Therefore, there is almost no environmental pollution caused by automobile transportation, and this external damage cost can be ignored.

The annual power generation of CS thermal power plant is approximately 5.64 million MWH; therefore, it can be concluded that the external damage cost per kilowatt hour in the raw material extraction process is 0.0024 yuan.

#### Current status of energy consumption and waste discharge in the electricity production process

In the electricity production process of CS thermal power plant, a large amount of energy costs are invested in fuel transportation, boiler combustion, steam turbine power generation, and waste treatment, mainly including coal, diesel, and electricity. The output products are electrical energy, with an annual output of 5.64 million MWH, which is transported to the urban area and surrounding areas of Changsha.

At the same time, the electricity production process also generates a large amount of waste, forming external damage costs, mainly including solid waste discharge pollution costs, wastewater discharge pollution costs, and exhaust gas discharge pollution costs.

1. Solid waste discharge pollution cost: The annual production of fly ash is 565,000 tons, with an environmental value of 0.12 yuan/kg. At the same time, due to the agreement signed between CS thermal power plant and the Coal Building Materials Industry Management Office, the cement plant near the power plant will recycle the generated fly ash, with a recovery price of 85 yuan/ton.
2. Cost of wastewater discharge pollution: The total annual discharge of wastewater from CS thermal power plant is 38,400 tons, with an environmental value of 0.0008 yuan/kg.
3. Cost of exhaust emissions and pollution: The annual emission of SO2 from CS thermal power plants is 2400 tons, with an environmental value of 6 yuan/kg; The annual emission of CO2 is 821,925.66 tons, with an environmental value of 0.09 yuan/kg; annual NOx emissions of 5680 tons. Table 5 shows the external damage costs of CS thermal power plant waste.

**Table 5:** External damage costs of CS thermal power plant waste

Cost items	Specific waste	Production volume (ton)	Environmental value (10,000 yuan)	Recycling price (10,000 yuan)	Net damage cost (10,000 yuan)
Solid waste discharge pollution cost	Fly ash	565,000.00	6780.00	4802.50	1977.50
Wastewater discharge pollution Cost	/	38,400.00	3.07	/	3.07
Emissions pollution cost	SO2	2400.00	1440.00	/	13,381.80
	CO2	821,925.66	7397.80	/	
	NOX	5680.00	4544.00	/	
In total	/	/	/	/	15,362.37

The evaluation system for low-carbon project investment in CS thermal power plants only includes the thermal power plant itself, only focusing on the economic benefits generated by the project for the thermal power plant, without considering the entire supply chain. Therefore, the selected low-carbon projects may not achieve the maximization of the overall benefits of the supply chain, which is not conducive to the sustainable development of the supply chain.

### Current investment status of low-carbon projects in CS thermal power plants

There are two types of low-carbon projects in general enterprises. One is to improve existing processes and technologies, or to improve existing equipment to improve product quality and achieve economic and environmental benefits; The second is to build projects to eliminate environmental pollution, by purchasing and installing certain cleaning equipment to treat environmental pollution generated by enterprises, reduce carbon emissions, and reduce external damage costs.

The low-carbon project chosen by CS thermal power plant for investment is based on the existing power generation capacity, utilizing mature, advanced, and applicable technologies to invest in and improve existing production equipment and auxiliary equipment, and upgrade control systems such as combustion management and regulation systems. This low-carbon project can be divided into four categories: boiler main equipment renovation project, steam turbine main equipment renovation project, water treatment system equipment renovation project, and auxiliary equipment renovation project. Due to the high management cost of production equipment in CS thermal power plants, the single product structure, and the need for updates and structural adjustments, investing in low-carbon projects aims to improve equipment performance, improve unit efficiency, reduce equipment costs, save energy and consumption, reduce carbon emissions, improve the environment, and enhance the comprehensive benefits of the supply chain. Table 6 shows the environmental protection investment projects and amounts.

**Table 6:** Environmental protection investment projects and amounts

Item	Cost (ten thousand yuan)
Circulating water pump motor pole change	100.00
Pulverized coal burner	105.00
Air feeder for the boiler	150.00
Continuous emission monitoring system	50.00
Wastewater discharge metering facilities	60.00
wastewater treatment facilities	250.00
In total	705.00

With the rise of new energy and the release of new national policies, the operating pressure of CS thermal power plants is gradually increasing, resulting in relatively less investment in low-carbon projects. Advanced energy-saving and consumption reducing new processes and technologies at home and abroad have not been fully applied, and there are still many problems in thermal power plants, such as high CO2 emissions from boilers; The thermal efficiency of the boiler is below 91% under different load interruptions, which is lower than the design value of 93.11%; The exhaust temperature of the boiler is relatively high, exceeding the design value by 10 ℃.

When CS thermal power plants invest in low-carbon projects, they still use traditional project investment evaluation methods, focusing on financial indicators such as investment payback period, investment return rate, and electricity saving income. This method can help enterprises choose projects with higher returns when making decisions, but it lacks social evaluation, does not fully consider the impact of the project on the environment, and ignores the feasibility of the project in the era of low-carbon economy.

## Design of low-carbon project investment evaluation for CS thermal power plants from the perspective of supply chain

In response to the problems in the investment evaluation of low-carbon projects in CS thermal power plants, this article starts from the perspective of low-carbon supply chain and improves the evaluation system throughout the entire process, in order to promote the attention and importance of power enterprises to low-carbon projects.

### Investment evaluation analysis of low-carbon projects at the supply chain level

#### Investment evaluation ideas for low-carbon projects at the supply chain level

The investment evaluation of low-carbon projects in this article is to calculate and evaluate the overall cost-effectiveness of the entire supply chain where CS thermal power plants are located, including economic cost-effectiveness, carbon cost-effectiveness, and collaborative benefits. For upstream coal mining enterprises, investing in low-carbon projects in CS thermal power plants may change the quantity of coal supply, affect the external damage costs of the supply chain in the mining process, and thus have an impact on the cost accounting of the project. For the downstream transmission and sales links, the data calculation in this article refers to Zhao Wenhuis carbon reduction investment decision model for the primary supply chain of electricity sales.

1 $${R}_{MAX}=\left(\alpha -\beta kQ+\beta D\right)\times Q+{P}_{CO2}\left(G-kQ+D\right)-C(Q)-C(D)$$

Among them, *P* = *α-βkQ*, P represents the retail price of electricity, *α* is a constant, *β* represents the sensitivity coefficient of consumer carbon footprint, *k* represents the unit carbon emission coefficient of power plants, and *Q* represents the electricity consumption;

$P_\textit{CO2}\left(G-kQ+D\right)$ represents carbon trading revenue;

$C\left(Q\right)$ represents the production cost of a power generation enterprise;

$C\left(\mathrm{D}\right)$ denotes the cost of emission reduction.

**Fig. 1:**
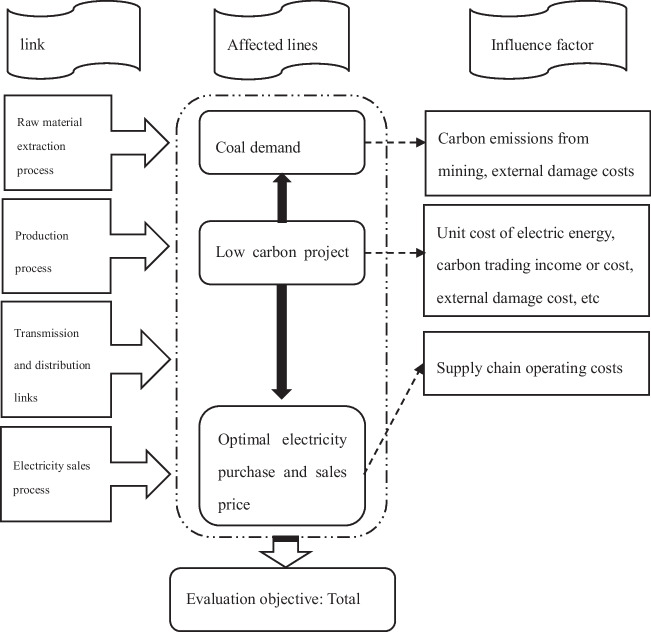
Investment evaluation ideas for low-carbon supply chain projects

In this model, the overall profit of the power supply chain can be optimized due to the optimal synergy between the power generation company and the power sales company. Therefore, this article aims to maximize the overall profit of the supply chain. By incorporating the data of investment in the power generation process into the model calculation and taking the partial derivative of the model equation with respect to Q and D to equal 0, the optimal equilibrium solution can be obtained to predict the data of the electricity sales process, including the optimal electricity purchase quantity and the optimal retail price of electricity. Moreover, this article only considers the cost of grid fees in the transmission process. By predicting the purchase of electricity in the sales process, the cost of the transmission process is equal to the grid fees multiplied by the purchase of electricity. Therefore, it will affect the overall cost of the transmission process, which can help this article evaluate the investment of CS thermal power plants at the supply chain level. Figure 1 shows the investment evaluation ideas for low-carbon supply chain projects.

#### Economic cost–benefit analysis of low-carbon project investment at the supply chain level

The traditional evaluation of enterprise project investment is generally biased towards projects with high economic returns. It mainly evaluates the economic indicators of each scheme, focusing on investment payback period, net present value, internal rate of return, investment rate of return, and profit index. When evaluating the investment in low-carbon projects at the supply chain level, in addition to considering the cost-effectiveness of thermal power plants, other cost benefits of the supply chain should also be considered when calculating the benefits. Not only traditional financial indicators should be evaluated, but also economic benefits indicators at the supply chain level should be included. When conducting cost accounting, based on the results of domestic and foreign literature, supply chain costs are divided into three categories: direct cost, activity cost, and transaction cost.

#### Carbon cost benefit analysis of low-carbon project investment at the supply chain level

The low-carbon supply chain theory indicates that all aspects of the supply chain involve energy consumption and carbon emissions, so all enterprises in the supply chain should work together to reduce emissions. In cost accounting, in addition to traditional costs, carbon costs are also considered. Currently, research on carbon accounting is developing. C﻿ombined with the actual situation of this case, carbon emission costs, testing costs, and contingent costs will be considered when calculating carbon costs.

At present, there is not much research on the evaluation of investment benefits at the supply chain level, but there are many studies on the performance evaluation of the supply chain, which can be used as a reference to some extent. Based on the opinions proposed by scholars and considering the special situation of the power supply chain, when evaluating projects, the overall carbon emissions reduction of the supply chain and the carbon trading income brought by carbon emissions reduction should be considered in terms of carbon benefits.

#### Collaborative benefit analysis of low-carbon project investment at the supply chain level

In the entire supply chain, due to project investment, it will affect the output, production costs, product energy consumption, etc. of the production enterprise. Based on the goal of maximizing profits, downstream enterprises will make certain adjustments, which will ultimately affect the collaborative ability of the supply chain. Therefore, it is necessary to conduct investment evaluation on the collaborative indicators of the supply chain. In supply chain management, collaboration indicators mostly come from supply chain performance research, mainly focusing on supply chain production and sales rate, supply chain production and demand rate, product production cycle time, inventory turnover rate, on time delivery rate, supply chain flexibility, low-carbon recognition, and low-carbon information sharing rate. However, in low-carbon projects of thermal power plants, the evaluation of supply chain collaboration benefits mainly involves whether supply and demand are balanced, assuming that the annual production of thermal power plants remains unchanged. Due to changes in the optimal purchasing power of electricity selling companies under different investment schemes, this indicator may vary among different schemes.

#### Summary of investment analysis for low-carbon projects at the supply chain level

Comparing the low-carbon project investment evaluation of low-carbon supply chain theory with traditional enterprise project investment evaluation, the expanded carbon cost-effectiveness, supply chain cost-effectiveness, and supply chain collaboration degree cannot be reflected in traditional investment benefit evaluation. When evaluating low-carbon project investments, a focus should be placed on reducing carbon emissions to establish an evaluation system. This not only focuses on the internal low-carbon evaluation of power plants, but also emphasizes controlling the overall carbon emissions of the supply chain, with the goal of maximizing supply chain profits. After investing in low-carbon projects at the power generation end, the seller should respond promptly to changes in the power generation end, which is also beneficial for power plants to adjust their production scale in a timely manner, maximizing profits while minimizing carbon emissions in the supply chain, achieving the goal of low-carbon production. Therefore, it is necessary to construct a low-carbon project investment benefit evaluation system based on the supply chain perspective.

**Fig. 2:**
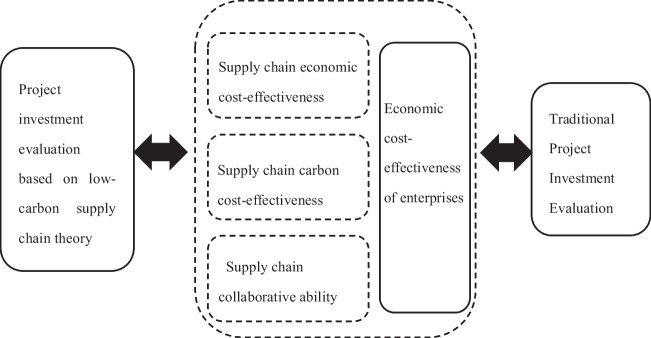
Differences in investment evaluation between traditional investment evaluation and supply chain perspective for low-carbon projects

Figure 2 defines the differences in investment evaluation between traditional investment evaluation and supply chain perspective for low-carbon projects.

**Fig. 3:**
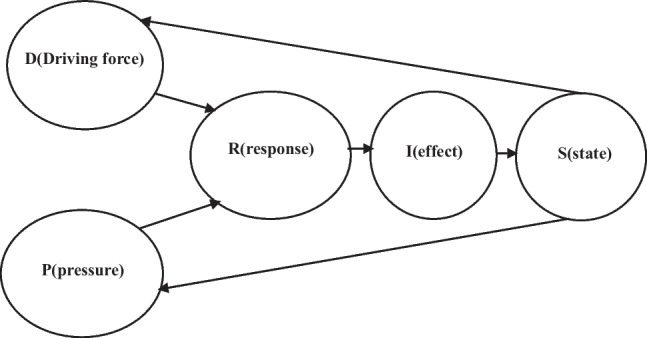
Based on the perspective of supply chain CS thermal power plant low-carbon project investment evaluation system mechanism diagram

Figure 3 shows the perspective of supply chain CS thermal power plant low-carbon project investment evaluation system mechanism diagram.

### Evaluation index system of CS thermal power plant low-carbon project investment from the perspective of supply chain

#### Analysis of low-carbon project investment evaluation mechanism based on DPSIR model framework

The DPSIR model is developed on the basis of the PSR model. It mainly analyzes the interaction between human activities and environmental systems. The analysis considerations mainly include five parts: driving force, pressure, state, impact, and response. This paper uses the DPSIR model to establish an index system. Relevant evaluation indicators are extracted from the two levels of the enterprise and the supply chain. These indicators are classified according to the model, so that there is a certain causal relationship between the indicators. Based on the investment evaluation of low-carbon projects in CS thermal power plants from the perspective of supply chain, the original DPSIR model mechanism is improved, and the state is taken as the evaluation target. The causal mechanism is as follows: driving force and pressure on the power supply chain cause enterprises in the power supply chain to respond and invest in low-carbon projects. In order to maximize profits and then influence the behavior of enterprises in the supply chain, the state of the entire power supply chain is changed, and the overall benefits of the supply chain are changed, including economic benefits and carbon benefits.

#### The evaluation index of low-carbon project investment in supply chain

In this paper, the investment of low-carbon projects in CS thermal power plants is taken as the research object, and the indicators are selected according to the cost–benefit analysis and the relevant policy requirements of the country and industry. The frequency statistics method, expert consultation method, and index principal component analysis method are used to select 13 evaluation indicators. Based on the DPSIR model framework, the evaluation indicators of low-carbon project investment in thermal power plants from the perspective of supply chain are as follows: Tables 3, 4, and Table 7 shows the CS thermal power plant supply chain level low-carbon project investment evaluation index.

**Table 7:** CS thermal power plant supply chain level low-carbon project investment evaluation index

Target layer	Indicator classification	Criterion layer (first level indicator)	Indicator layer (secondary indicator)
Evaluation index system for low-carbon project investment at the supply chain level of CS thermal power plants	Result metrics	S (status)	Supply chain production and demand rate S1
			Overall profit growth rate of the supply chain S2
			Carbon dioxide reduction S3
	Impact indicators	D (drive)	Investment return rate D1
			Carbon trading revenue or cost D2
		P (pressure)	Total operating cost of the supply chain P1
			Unit production cost P2
			Energy consumption per unit output value P3
			External damage cost P4
			Investment payback period P5
		R (respond)	Low-carbon technology investment R1
		I (influence)	Optimal purchasing power I1
			Optimal retail price for electricity I2

##### State

The state is the result of the combined action of driving force and pressure, indicating the state of the supply chain after investing in low-carbon projects.

- 1) Supply chain production and demand rate S1.
  2 $$Supply\;chain\;production\;and\;demand\;rate=power\;plant\;power\;output / power\;sales\;enterprise\;demand\;for\;electric\; energy\;products$$
  If the index value is greater than 1 or equal to 1, it shows that the overall operation of the power supply chain is good, the production capacity is strong, and the market competitiveness is strong. If the index is less than 1, it shows that the power supply chain also needs to strengthen production capacity and needs to accelerate the response to market demand.
- 2) The overall profit growth rate of supply chain S2.
  The formula of the overall profit growth rate of the supply chain is as follows:
  3 $$P=\alpha -\beta kQ$$
  In the above formula, *P* represents the retail price of electricity; *α* is a constant; *β* represents the consumer carbon footprint sensitivity coefficient; *k* represents the unit carbon emission coefficient of the power plant that is t/KW h; *Q* represents the electricity purchased by the electricity retailer.
  The profit function of power supply chain is:
  4 $$R=\left(P-F\right)\text{Q}+P_\textit{CO2}\left(G-kQ\right)-C\left(Q\right)$$
  In the above formula, *P* represents the retail price of electricity; *F* represents the network service fee, the unit is yuan/KW·h; ${P}_{CO2}$ denotes the revenue or cost of carbon trading, where G denotes the carbon emission limit given by the government;
  $C\left(Q\right)$ indicates the production cost function of a power plant:
  5 $$C\left(Q\right)=bQ\left(\mathrm{Production\;cost\;per\;unit}\right)$$
  The profit after investing in low-carbon projects is expressed by $R_\text{MAX}$, which also indicates the maximum profit that the power supply chain can obtain in investing in low-carbon projects.
  6 $$R_{MAX}=\left(\alpha-\beta kI1+\beta S3\right)\times I1+P_\textit{CO2}\left(G-kI1+S3\right)-C\left(Q\right)-C\left(S3\right)$$
  In the above formula, $I{1}$ is the optimal electricity purchase index, $C(\mathrm{S}{3})$. The optimal electricity purchase index represents the input of low-carbon technology, that is, the index *R*1:
  7 $$C\left(S{3}\right)=\varepsilon {S{3}}^{2} \left(\varepsilon \mathrm{Cost\;coefficient\;of\;carbon\;emission\;reduction}\right)$$
  So:
  8 $$I1=R_\textit{MAX}-R$$
- 3) Emissions reduction of CO2 S3.

This indicator is expressed in tons.

The formula for calculating carbon dioxide emissions is recommended by the United Nations Climate Expert Committee (IPCC):

9 $$C=(Q\times M-F)\times S\times \frac{44}{12}$$

In the above formula, *Q* refers to energy consumption; *M* is the carbon contained in unit energy; *F* is the amount of carbon sequestration in enterprise production; *S* is the oxidation rate in the process of energy utilization. (A ton of carbon can produce about 3.67 tons of carbon dioxide after burning in oxygen, because the molecular weight of C is 12 and the molecular weight of CO2 is 44, 44/12 = 3.67).

So:

10 $${\text{S}}{3}={\text{C}}{\text{F}}-{\text{C}}{\text{P}}$$

In the above formula, *CF* represents carbon dioxide emissions after investment in low-carbon projects, and *CP* represents carbon dioxide emissions before investment in low-carbon projects.

Referring to the formula recommended by IPCC, combined with the actual situation of this case, the carbon dioxide emission reductions in this paper mainly include coal-saving emission reductions and electricity-saving emission reductions. The calculation of the index is listed as follows:

11 $$C={Q}_{\alpha }\times K$$

In the above formula, *C* represents carbon dioxide emissions in tons; *Qα* represents the amount of energy used, converted to the value of standard coal; *K* indicates that the carbon dioxide emission coefficient of energy use after standard coal is calculated (according to the data of the carbon emission trading network, the carbon dioxide emission coefficient of coal is 3.67, and the carbon dioxide emission coefficient of 1 degree electricity is 0.997 because 1 degree electricity = 0.4 kg standard coal).

##### Driving force

The driving force is the power source for enterprises to invest in low-carbon projects.

- 1) Return on investment D1
  12 $$Return\;on\;investment = total\;profit\;/\;total\;investment\;in\;low-carbon\;projects\times\;100\%$$
- 2) Carbon transaction costs or income D2.

The index is in ten thousand yuan as a unit.

13 $$Carbon\;transaction\;cost\;or\;income = | actual\;carbon\;emissions - rated\;carbon\;emissions | \times\;carbon\;trading\;price$$

In this paper, the index measures the additional income brought by carbon dioxide emission reduction, which is obtained by multiplying carbon emission reduction by carbon trading price. According to the data of carbon emission network and referring to Hubei carbon trading market, the carbon trading price is 31.78 yuan/ton.

##### Stress

- 1) Total operating cost of supply chain P1. The operating cost of the power supply chain mainly refers to the cost of electricity transportation. The greater the electricity purchased by the electricity retailers, the higher the operating cost. In addition, the operating costs of this article also include the operating costs of low-carbon projects; refer to the project put into use, in order to maintain the normal operation of the project, the resulting equipment maintenance costs, labor costs, and equipment depreciation costs, to ten thousand yuan as a unit.
- 2) Unit production cost P2. The unit production cost is the cost of the average consumption of the production unit product, in yuan.
  14 $$unit\;cost\;of\;production=C(Q)/Q$$
  $${\text{Q}}\text{ denotes the amount of electricity purchased by the electricity selling company.}$$
- 3) Energy consumption per unit output value P3
  15 $$Bit\;output\;value\;energy\;consumption=E/G$$
  In the above formula, *E* represents the total energy consumption of electric energy production after the low-carbon project is put into operation, which is converted into tons of standard coal; *G* represents the net output value of electric energy production in a certain period, in yuan.
- 4) External damage cost reduction P4. External damage cost in this paper refers to the cost of environmental damage caused by carbon dioxide emissions in the process of electric energy production, plus the cost of external damage to forests in the process of coal mining. The external damage cost index of carbon dioxide is calculated by referring to the LIME coefficient table. This paper uses the exchange rate at the end of 2018 to convert the LIME value (yen) into RMB.
  16 $$External\;damage\;cost = carbon\;dioxide\;emissions\;\times\;LIME\;coefficient + coal\;consumption\;\times\;unit\;forest\;value$$
  17 $$External\;damage\;cost\;reduction = external\;damage\;cost\;after\;investment-external\;damage\;cost\;before\;investment$$
- 5) Investment recovery period P5.

The calculation formula of static investment payback period is as follows:

18 $$\sum\nolimits_{t=1}^{{\text{P}}{3}}{\text{R}}{\text{t}}=A$$

In the above formula, *A* represents the initial investment amount, *Rt* refers to the net income of the *t* year after the investment of the project, and *P3* is the final investment recovery period.

##### Response

Low-carbon technology investment P2. This indicator refers to the total investment in low-carbon projects.

##### Influence

The influence index mainly provides the basis for the calculation of the state index, which is not included in the calculation of the evaluation model.

- 1) The optimal electricity purchase quantity I1.
  In order to maximize the profit *R* of the power supply chain, the optimal power purchase S5 can be obtained from the calculated *P*; that is, the optimal equilibrium solution of *Q* is:
  19 $${I}_{1}=\left[2\varepsilon \times \left(\alpha -F-b\right)-{P}_{CO2}\times \left(2k\varepsilon -\beta \right)\right]/\left(4k\varepsilon -\beta \right)\times \beta$$
  In the above formula, *ε* is the cost coefficient of carbon emission reduction; *α* is a constant; *F* represents the network service fee; the unit is yuan/KW h; *b* is the unit production cost; *β* represents the consumer carbon footprint sensitivity coefficient; *k* represents the unit carbon emission coefficient of the power plant is t/KW h; $P_\textit{CO2}$ represents the carbon trading price.
- 2) The optimal price of electricity I2.

In order to maximize the profit *R* of the power supply chain, the optimal retail price of electricity can be obtained as S4; that is, the equilibrium solution of the optimal *P* is:

20 $${P}=[3{k\varepsilon \alpha }+({F}+{b})\times (2{k\varepsilon }-\upbeta )/4{k\varepsilon }-\upbeta +2{k}^{2}\varepsilon \times {P}_{CO2}]/4k\upvarepsilon -\upbeta$$

In the above formula, *k* represents the unit carbon emission coefficient of the power plant which is t/KW h; *ε* is the cost coefficient of carbon emission reduction; *β* represents the consumer carbon footprint sensitivity coefficient; *f* represents the network service fee, the unit is yuan/KW h; *b* is unit production cost; $P_\textit{CO2}$ represents the carbon trading price.

### Construction of CS thermal power plant low-carbon project investment evaluation model from the perspective of supply chain

#### Selection of evaluation methods

The purpose of constructing the evaluation index from the perspective of supply chain is to evaluate the overall efficiency of the supply chain of enterprises investing in low-carbon projects. Therefore, after a comprehensive comparison of various methods, this paper first uses the analytic hierarchy process (AHP) to determine the weight of the established low-carbon project investment evaluation index at the supply chain level. Secondly, the gray correlation analysis method is selected to evaluate the low-carbon project plan. The main reason is that the case of this paper is in line with the characteristics of the grey system. The essence of the investment evaluation of low-carbon projects in power generation enterprises in this paper is to find a project plan that can maximize the benefits of the supply chain based on the analysis of the supply chain situation, with the goal of economic benefits and carbon benefits. Due to the limited information of the project plan in the case, and based on the power supply chain model, the power generation end data is used to predict the power sales end data, so as to obtain the overall data of the supply chain. The sample information data is insufficient. Therefore, this paper uses the grey correlation analysis method to evaluate the low-carbon project plan of CS power plant and select the optimal plan.

#### The analytic hierarchy process to determine the index weight

**Fig. 4:**
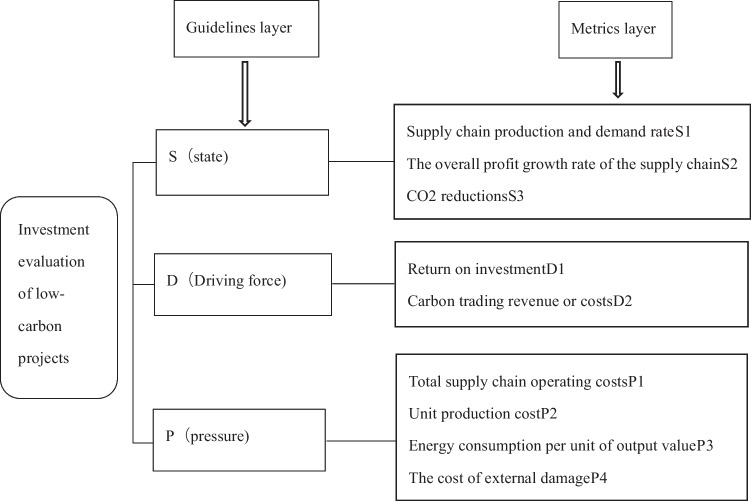
Low-carbon project investment evaluation hierarchy diagram

The analytic hierarchy process is used to determine the index weight. The main process includes the following steps:

- (1) Establish hierarchical structure model. The index is divided into two levels: criterion layer, including pressure, driving force, state, influence, and response in DPSIR model framework; it refers to the surface layer, including 13 specific indicators. However, due to the particularity of the DPSIR model framework, in order to construct a complete causal relationship between the indicators, not all indicators will be included in the calculation of the final results. The optimal electricity purchase I1, the optimal electricity retail price I2, and the low-carbon technology input R1 belong to the process indicators, which will affect the values of other indicators and are only used in the calculation of other indicators. Therefore, these three indicators will be ignored in the measurement of the results, and only the other 10 indicators will be considered.
  Figure 4 shows the low-carbon project investment evaluation hierarchy diagram.
- (2) Constructing judgment matrix
  The judgment matrix is constructed for the index of the criterion layer and the index of the surface layer. The scale method of 1–9 and its reciprocal is used to compare the importance between the indexes, and the expert is invited to give the judgment result. Table 8 shows the scale of relative importance of elements. Table 9 shows the criterion layer judgment matrix. Table 10 shows the state index judgment matrix. Table 11 shows that the driving force index judgment matrix. Table 12 shows the pressure index judgment matrix.
- (3) Calculating judgment matrix
  Matlab software is used to calculate the maximum eigenvalue of the judgment matrix and its corresponding eigenvector, and the weight coefficient distribution of the low-carbon project investment evaluation index.

**Table 8:** Scale of relative importance of elements

Scale	Meaning
1	Two elements are equally important compared to each other
3	The former is slightly more important than the latter
5	The former is quite important compared to the latter
7	The former is significantly more important than the latter
9	The former is absolutely more important than the latter
2, 4, 6, 8	Intermediate value of adjacent judgments mentioned above
Count backwards	If the aij of element i is compared to element j, then the ratio of element j to element i is 1/aij

**Table 9:** Criterion layer judgment matrix

Index	S	D	P
S (status)	1		
D (drive)		1	
P (pressure)			1

**Table 10:** State index judgment matrix

Status indicators	S1	S2	S3
Supply chain production and demand rate S1	1		
Supply chain profit growth rate S2		1	
Carbon dioxide reduction S3			1

**Table 11:** Driving force index judgment matrix

Driving force indicators	D1	D2
Investment return rate D1	1	
Carbon trading revenue or cost D2		1

**Table 12:** Pressure index judgment matrix

Pressure indicators	P1	P2	P3	P4	P5
Total operating cost of the supply chain P1	1				
Unit production cost P2		1			
Energy consumption per unit output value P3			1		
External damage cost P4				1	
Investment payback period P5					1

- (4) Consistency test

After the feature vector is calculated, the consistency test of the judgment matrix is carried out. When the random consistency ratio is calculated, the consistency test of the judgment matrix is carried out. When CR equals CI divided by RI, and the value is less than 0.10, the judgment matrix is valid and the weight distribution is reasonable.

The test coefficient CR is determined by the ratio of CI to RI. And Table 13 shows the average random consistency of index.

**Table 13:** Average random consistency index

*n*	1	2	3	4	5	6	7	8	9	10	11	12	13	14	15
RI	0	0	0.58	0.90	1.12	1.24	1.32	1.41	1.45	1.49	1.51	1.54	1.56	1.58	1.59

21 $$CI=\frac{{\lambda }_{\mathrm{max} }-n}{n-1}$$

#### Grey correlation analysis to determine the evaluation results

The basic steps of grey correlation analysis are as follows:

- (1) Initialization of the original data
  In the investment evaluation index of low-carbon projects, due to the differences in the meaning and value criteria of different indicators, some indicators are positive indicators, and some indicators are negative indicators. In order to make them comparable, the data should be dimensionless, so that these indicators can be uniformly measured.
  When the index is a positive index, the standardized formula is:
  22 $${x}_{ij}{\prime}=\frac{{x}_{ij}}{{x}_{j}^{\mathrm{max}}}$$
  When the index is negative, the standardized formula is:
  23 $${x}_{ij}{\prime}=1-\frac{{x}_{ij}}{{x}_{j}^{\mathrm{max}}}$$
- (2) Calculate the absolute difference between the comparison sequence and the reference sequence
  24 $$\begin{array}{cc}{\left(\left|{\mathrm{x}}_{\mathrm{ij}}-{\mathrm{x}}_{0\mathrm{j}}\right|={\Delta }_{0\mathrm{j}}\left(j\right)\right)}_{\mathrm{n}\times \mathrm{p}}& \begin{array}{cc}i=\mathrm{1,2},\dots ,n& \mathrm{j}=\mathrm{1,2},\dots ,p\end{array}\end{array}$$
  The comparison sequence refers to the original value of each index. The reference sequence is constructed by selecting the optimal value from the original values of each index in several schemes. When the index is a positive index, the maximum value of the index in each scheme is optimal.
- (3) Calculate the grey coefficient of the index system
  In the grey correlation analysis method, the correlation coefficient is the distance between the reference sequence and the comparison sequence, that is, the gap between the index value and the optimal value. The larger the value of the correlation coefficient, the greater the correlation between the index and the optimal value.
  25 $$\begin{array}{cc}{\zeta }_{0i}(j)=\frac{\mathrm{min}{\Delta }_{0i}(j)+\rho \mathrm{max}{\Delta }_{0i}(j)}{{\Delta }_{0i}(j)+\rho \mathrm{max}{\Delta }_{0i}(j)}& 0<\rho <1\end{array}$$
  where $\rho$ is a constant, usually, $\rho$ takes 0.5.

- (4) Calculate the comprehensive grey correlation degree and arrange the correlation order

Because low-carbon project investment evaluation indicators have multiple indicators, there are many correlation coefficients, and the distribution is scattered. The comprehensive grey correlation degree is to concentrate these correlation coefficients and reflect the correlation degree of the evaluation index series of low-carbon project investment in general. The larger the value of grey correlation degree, the better.

The calculation formula of comprehensive grey correlation degree is obtained by weighted average of grey correlation coefficient and analytic hierarchy process weight:

26 $$r{\text{0i}}=\sum\nolimits_{j=1}^{p}\omega {\text{j}}\zeta {\text{0j}}\left(j\right) i=1,\dots ,n$$

## The implementation and application analysis of CS thermal power plant low-carbon project investment evaluation system

### Program of investment

At present, there are the following problems in the operation of CS thermal power plant: first, the operation of circulating water pump is affected by dry season and wet season, water temperature, and so on, so that the coal consumption of power generation is high; secondly, the boiler thermal efficiency is lower than 91% under different load interruptions, which is lower than the design value of 93.11%. Third, the boiler exhaust gas temperature is higher, exceeding the design value of 10 °C, resulting in higher coal consumption. In order to solve this series of problems, some low-carbon projects are ready to be put into operation. However, due to the limited short-term funds, a priority investment plan needs to be selected. The investment plans of these projects are as follows:

#### Scheme 1

Circulating water pump motor pole transformation project. Nowadays, technology can be used to slow down the motor without adjusting the speed to increase power consumption, increase the amount of coal, resulting in increased production costs. In the DC water supply system, the 16-pole motor of the circulating water pump is transformed into a 16/18-pole motor, and the power of the circulating water pump motor is changed from 3800 to 3800/2500 kW. The working mechanism of the circulating water pump motor is adjusted from the original single-speed mode to the dual-speed mode, and then continues to be subject to the above various influencing factors. By changing the working speed of the motor to control the output water volume, the purpose of allowing the turbine to operate in a near-vacuum environment can be achieved. In this way, energy consumption can be effectively reduced. The investment amount of the project is 1 million yuan, the construction period interest is 238 thousand yuan, the investment recovery period is 0.4 years, the annual saving of coal for power generation is 2614.5 tons, the annual saving of electricity is 128,700 KWh project, and the total expected profit is 2.1391 million yuan.

#### Scheme 2

Coal burner renovation project. At present, the boiler burner is a direct-flow burner. The outstanding problem of this traditional direct-flow burner is that the temperature in the furnace is not high, which often causes the boiler to flame out. In this project, the direct-flow–pulverized coal burner in the traditional pulverized coal furnace is replaced by a stable combustion burner with louvered windows installed at the nozzle, which can greatly ensure the stable combustion at low load and the durability of the nozzle. Table 14 shows the economic indicators and energy saving effect of each investment project. Table 15 shows the comparison of environmental benefits of each project. Table 16 shows the data table of different project schemes. Table 17 shows the criterion layer judgment matrix. Table 18 shows the rule layer weight table. Table 19 shows the state index judgment matrix. Table 20 shows the state index weight table. Table 21 shows the driving force index judgment matrix. Table 22 shows the driving force index weight table. Table 23 shows the pressure index judgment matrix. Table 24 shows the pressure index weight table. Table 25 shows the weight of each index. Table 26 shows the index dimensionless results table. Table 27 shows the absolute difference table. Table 28 shows the grey coefficient table. Table 29 shows the scheme score table.

**Table 14:** Economic indicators and energy saving effect of each investment project

Item	Pole changing transformation of circulating water pump motor	Transformation of coal powder burner	Energy saving renovation of boiler air supply fan
Investment amount (10,000 yuan)	100.00	105.00	150.00
Interest during construction period (10,000 yuan)	2.38	6.47	16.85
Investment payback period (years)	0.40	1.32	2.40
Annual energy savings (KWh)	128,700	/	337,500.00
Annual coal savings for power generation (tons)	2614.50	4821.61	/
Estimated total profit (10,000 yuan)	213.91	351.64	435.78

**Table 15:** Comparison of environmental benefits of each project

Item	Pole changing transformation of circulating water pump motor	Transformation of coal powder burner	Energy saving renovation of boiler air supply fan
Annual resource saving limit (10,000 yuan)	136.35	241.08	14.75
CO2 emissions from the mining process (tons)	436,800.00	436,800.00	436,800.00
CO2 emissions from production processes (tons)	821,925.66	821,925.66	821,925.66
Emission reduction (ton)	235,900.00	215,800.00	223,310.00
Total CO2 emissions after investment (tons)	1,022,800.00	1,042,900.00	1,035,600.000
Cost of external forest damage during mining (10,000 yuan)	1396.35	1396.35	1396.35
External damage cost of carbon emissions in the mining process (10,000 yuan)	3931.20	3931.2	3931.20
External damage cost in production process (10,000 yuan)	15,362.37	15,362.37	15,362.37
Reduced cost of external damage to the supply chain (10,000 yuan)	2124.50	1944.79	2007.90
Total external damage cost of the supply chain after investment (10,000 yuan)	18,565.42	18,745.13	18,682.02

**Table 16:** Data table of different project schemes

Index	Scheme 1	Scheme 2	Scheme 3
Supply chain production and demand rate S1	1.69	2.06	1.55
Overall profit growth rate of the supply chain S2	2.06	1.80	1.88
Carbon dioxide reduction S3	23.59	21.58	22.33
Investment return rate D1	2.13	3.35	2.61
Carbon trading revenue or cost D2	749.69	685.81	709.01
Total operating cost of the supply chain P1	386.28	338.10	355.62
Unit production cost P2	0.36	0.37	0.36
Energy consumption per unit output value P3	306.80	308.80	307.30
External damage cost P4	18,565.42	18,745.13	18,682.02
Investment payback period P5	0.40	1.32	2.40

**Table 17:** Criterion layer judgment matrix

Indicators	S	D	P
S (state)	1	2	4
D (driving force)	½	1	2
P (pressure)	¼	½	1

**Table 18:** Rule layer weight table

Criterion layer	Weight
S (state)	0.57
D (driving force)	0.29
P (pressure)	0.14

**Table 19:** State index judgment matrix

State indicators	S1	S2	S3
Supply chain production and demand rate S1	1	1/3	1/2
Supply chain overall profit growth rate S2	3	1	2
Carbon dioxide emission reduction S3	2	½	1

**Table 20:** State index weight table

Index layer	Weight
Supply chain production and demand rate S1	0.16
Supply chain overall profit growth rate S2	0.54
Carbon dioxide emission reduction S3	0.30

**Table 21:** Driving force index judgment matrix

Driving force index	D1	D2
Return on investment D1	1	2
Carbon trading revenue or cost D2	½	1

**Table 22:** Driving force index weight table

Index layer	Weight
Return on investment D1	0.67
Carbon trading revenue or cost D2	0.33

**Table 23:** Pressure index judgment matrix

Pressure index	P1	P2	P3	P4	P5
Total supply chain operating cost P1	1	3	2	2	5
Unit production cost P2	1/3	1	1/3	1/3	2
Energy consumption per unit output value P3	1/2	3	1	1/2	3
External damage cost P4	1/2	3	2	1	4
Investment recovery period P5	1/5	½	1/3	1/4	1

**Table 24:** Pressure index weight table

Index layer	Weight
Total supply chain operating cost P1	0.37
Unit production cost P2	0.11
Energy consumption per unit output value P3	0.19
External damage cost P4	0.27
Investment recovery period P5	0.06

**Table 25:** Weight of each index

Target layer	Indicator classification	Criterion layer (first level indicator)	Weight	Indicator layer (secondary indicator)	Weight	Comprehensive weight
CS thermal power plant low-carbon project investment evaluation index system for supply chain	Result metrics	S (status)	0.57	Pressure supply chain production and demand rate S1	0.16	0.09
				Overall profit growth rate of the supply chain S2	0.54	0.31
				Carbon dioxide reduction S3	0.30	0.17
	Impact indicators	D (drive)	0.29	Investment return rate D1	0.67	0.19
				Carbon trading revenue or cost D2	0.33	0.10
		P (pressure)	0.14	Total operating cost of the supply chain P1	0.37	0.05
				Unit production cost P2	0.11	0.01
				Energy consumption per unit output value P3	0.19	0.03
				External damage cost P4	0.27	0.04
				Investment payback period P5	0.06	0.01

**Table 26:** Index dimensionless results table

Indicators	Scheme 1	Scheme 2	Scheme 3
Supply chain production and demand rate S1	1.00	0.87	0.92
Supply chain overall profit growth rate S2	1.00	0.87	0.91
Carbon dioxide emission reduction S3	1.00	0.91	0.95
Return on investment D1	0.64	1.00	0.78
Carbon trading revenue or cost D2	1.00	0.91	0.95
Total supply chain operating cost P1	0.00	0.12	0.08
Unit production cost P2	0.03	0.00	0.01
Energy consumption per unit output value P3	0.01	0.00	0.01
External damage cost P4	0.00	0.12	0.08
Investment recovery period P5	0.83	0.45	0.00

**Table 27:** Absolute difference table

Index	Scheme 1	Scheme 2	Scheme 3
Supply chain production and demand rate S1	0.00	0.13	0.08
Supply chain overall profit growth rate S2	0.00	0.12	0.09
Carbon dioxide emission reduction S3	0.00	0.08	0.05
Return on investment D1	0.36	0.00	0.22
Carbon trading revenue or cost D2	0.00	0.08	0.054
Total supply chain operating cost P1	0.12	0.00	0.05
Unit production cost P2	0.00	0.02	0.02
Energy consumption per unit output value P3	0.00	0.01	0.00
External damage cost P4	0.12	0.00	0.04
Investment recovery period P5	0.00	0.38	0.83

**Table 28:** Grey coefficient table

Indicators	Scheme 1	Scheme 2	Scheme 3
Supply chain production and demand rate S1	1.00	0.76	0.83
Supply chain overall profit growth rate S2	1.00	0.76	0.82
Carbon dioxide emission reduction S3	1.00	0.83	0.88
Return on investment D1	0.53	1.00	0.65
Carbon trading revenue or cost D2	1.00	0.83	0.88
Total supply chain operating cost P1	0.76	1.00	0.90
Unit production cost P2	1.00	0.93	0.95
Energy consumption per unit output value P3	1.00	0.98	0.99
External damage cost P4	0.76	1.00	0.90
Investment recovery period P5	1.00	0.52	0.33

**Table 29:** Scheme score table

Scheme	Comprehensive score
Scheme 1	0.88
Scheme 2	0.85
Scheme 3	0.81

### Application of evaluation system

#### Calculate the original value of evaluation data

According to the index calculation method, the original values of the 10 indexes calculated by the final evaluation model are obtained.

#### Determination of index weight

According to the low-carbon project investment evaluation index system constructed in this paper, through the questionnaire survey of expert consultation method, 8 experts in this field are selected to score the importance of the indicators, respectively. This paper constructs four judgment matrices of criterion layer and index layer and uses AHP-subjective and objective comprehensive assignment method to empower and test the consistency of its indicators.

- 1) Criterion layer matrix and consistency test
  The maximum eigenvalue of the judgment matrix is ${\lambda }_{\mathrm{max}}=\text{3.0000}$ calculated by MATLAB software. In order to test the consistency of the judgment matrix, it is necessary to calculate the consistency index:
  26 $$CI=\frac{{\lambda }_{\mathrm{max}}-n}{n-1}=\frac{\text{3.0000}-3}{3-1}=0$$
  Average random consistency index RI = 0.58. Random consistency ratio:
  27 $$CR=\frac{CI}{RI}=\frac{0}{0.58}=0<0.10$$
  Therefore, it is considered that the results of analytic hierarchy process are satisfactory; that is, the distribution of weight coefficients is very reasonable.
- 2) Index layer matrix and consistency test

Firstly, the judgment matrix of S (state) index is constructed.

28 $$S={\left({u}_{ij}\right)}_{p\times p}$$

The maximum eigenvalue of the judgment matrix is calculated by MATLAB software. *λ*_max_ = 3.0092. In order to test the consistency of the judgment matrix, it is necessary to calculate the consistency index:

29 $$CI=\frac{{\lambda }_{\mathrm{max}}-n}{n-1}=\frac{\text{ 3.0092}-3}{3-1}=\text{0.0046}$$

Mean random consistency index, RI = 0.58. Random consistency ratio:

30 $$CR=\frac{CI}{RI}=\frac{0.0046}{0.58}=\text{0.0079}<0.10$$

Therefore, it is considered that the results of the analytic hierarchy process are satisfactory; that is, the distribution of the weight coefficient is very reasonable, and the weight table is obtained.

Then, the judgment matrix of D (driving force) index is constructed.

31 $$S={\left({u}_{ij}\right)}_{p\times p}$$

The maximum eigenvalue of the judgment matrix is calculated by MATLAB software. *λ*_max_ = 2. Since there are only two indicators, the two-order matrix does not need to perform a consistency test, and the weight table is directly obtained.

Finally, the judgment matrix of P ( pressure) index is constructed.

32 $$S={\left({u}_{ij}\right)}_{p\times p}$$

The maximum eigenvalue of the judgment matrix is calculated by MATLAB software *λ*_max_ = 5.1223. In order to test the consistency of the judgment matrix, it is necessary to calculate the consistency index:

33 $$CI=\frac{{\lambda }_{\mathrm{max}}-n}{n-1}=\frac{\text{5.1223}-5}{5-1}=\text{0.0306}$$

Mean random consistency index RI = 1.12. Random interconsistency ratio:

34 $$CR=\frac{CI}{RI}=\frac{ 0.0306}{1.12}=\text{0.0273}<0.10$$

Therefore, it is considered that the results of the analytic hierarchy process are satisfactory; that is, the distribution of the weight coefficient is very reasonable, and the weight table is obtained.

According to the above calculation of the index weight in each index dimension, the weight and comprehensive weight of each index in the low-carbon project evaluation index system relative to the upper index can be summarized, as shown in the table.

#### The scheme results based on grey correlation analysis

First of all, in order to make the indicators comparable, the dimensionless processing of each indicator data is carried out to eliminate the respective effective factors of each data and make it a standardized order of magnitude dimensionless data under a unified measurement scale.

The dimensionless results are shown in Table 26.

Then, calculate the absolute difference between the comparison sequence and the reference sequence:

The grey coefficient of the index system is calculated as follows:

35 $$\begin{array}{cc}{\zeta }_{0i}(j)=\frac{\mathrm{min}{\Delta }_{0i}(j)+\rho \mathrm{max}{\Delta }_{0i}(j)}{{\Delta }_{0i}(j)+\rho \mathrm{max}{\Delta }_{0i}(j)}& 0<\rho <1\end{array}$$

where $\rho$ is a constant, usually, $\rho$ takes 0.5; this article $\rho$ is equal to 0.5.

The grey correlation degree is calculated and the correlation order is arranged. The grey correlation coefficient and the weight of the analytic hierarchy process are weighted and averaged to obtain the comprehensive grey correlation degree.

According to the comprehensive score, scheme 1 is the best and the comprehensive benefit is the best. Therefore, in the investment evaluation of low-carbon projects at the supply chain level, option 1 should be selected to invest in the transformation project of circulating water pump motor.

### Case summary

In view of the above case analysis and results, this paper puts forward the following suggestions for the investment evaluation of low-carbon projects in CS thermal power plants.

- (1) Focus on policy background and market changes
  CS power plant should pay attention to relevant national policies in time. On the issue of energy conservation and emission reduction, first of all, it is necessary to do a good job in emission reduction measures and reduce carbon expenditure according to policy requirements. Secondly, it is necessary to turn passive into active. As an enterprise with high energy consumption and high emission, it should actively integrate into the construction of energy conservation and emission reduction.
  In addition, the implementation of the No. 9 document also fully reflects the country’s determination to accelerate the reform of the electricity market. In the market-oriented reform of electricity, with free competition in price as the core, competition is introduced in the power generation and sales links, so as to open up both sides and control the middle. At the same time, with the development of power grid and energy storage industry as the support, the construction of infrastructure is strengthened, and a new type of electricity market is constructed. And once the market-oriented power system is formed, it will stimulate the innovation and development of the power industry from the demand side, change the current market environment, and ultimately affect the entire supply chain and form a new business model. Therefore, if CS thermal power plants want to gain a competitive advantage in the market, they will inevitably be affected by power sales companies and even consumers, so supply chain management must be strengthened.
- (2) Enhance the carbon management awareness of enterprises
  The management of the power plant should have a sense of carbon management, attach great importance to the carbon emission of the enterprise, control the carbon cost for each link of the production of the enterprise, find out the link of high energy consumption and high emission, as the entry point of its low-carbon project investment, and draw up a low-carbon project investment plan suitable for itself.
  In the evaluation of project investment, environmental costs and environmental benefits should be considered, carbon factors should be taken into account, environmental pollution caused by carbon emissions should be considered, and the benefits of energy conservation and emission reduction should be considered, and the low-carbon sensitivity coefficient of buyers should be considered. When purchasing products, the value of low-carbon investment of enterprises should be valued, and the best choice for the benefit of the whole supply chain should be made.
- (3) Establish project investment evaluation system at the supply chain level.

**Fig. 5:**
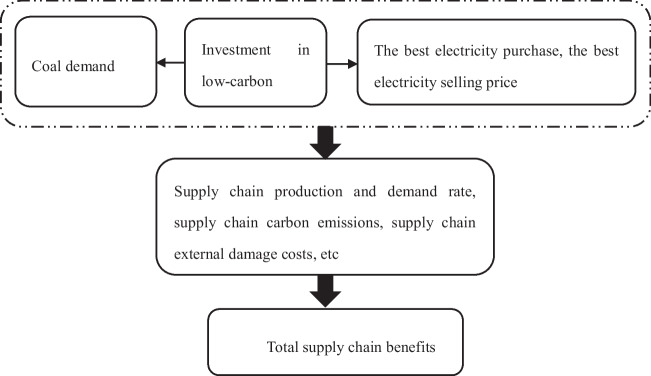
Supply chain impact route of low-carbon project investment

After the power reform, the competition in the power market has become larger. In the current supply chain era, enterprises need to cooperate with upstream and downstream enterprises in the supply chain. The investment behavior of individual enterprises will also affect other nodes of the supply chain. Therefore, the new project investment evaluation should take into account the supply chain level. In the evaluation of low-carbon project investment in thermal power plants, project investment will affect the output, production cost, and energy consumption of production enterprises. Based on the goal of profit maximization, downstream enterprises will make certain adjustments, which will ultimately affect the overall efficiency of the supply chain. Therefore, it is necessary to add the indicators of the supply chain to the evaluation system of low-carbon project investment. Considering the low-carbon projects of enterprises from the perspective of supply chain can make the project evaluation system of low-carbon power supply chain more scientific. Figure 5 shows the supply chain impact route of low-carbon project investment.

## Conclusion

Based on the theoretical basis of low-carbon supply chain, this paper extends the traditional enterprise project investment evaluation to the low-carbon project investment evaluation at the supply chain level, designs a set of low-carbon project investment evaluation system suitable for power enterprises from the perspective of supply chain, and overcomes the traditional emphasis on economic benefits without fully considering the disadvantages of carbon factors. The analytic hierarchy process and grey relational analysis are used to evaluate the low-carbon project investment of CS thermal power plant supply chain, verify the effectiveness of the evaluation system, and explore the new path of project investment evaluation from the perspective of supply chain.

## Data Availability

The data can be available on request.
